# Disability, Home Physical Environment and Non-Fatal Injuries among Young Children in China

**DOI:** 10.1371/journal.pone.0037766

**Published:** 2012-05-18

**Authors:** Hui-ping Zhu, Xin Xia, Hui-yun Xiang, Chuan-hua Yu, Yu-kai Du

**Affiliations:** 1 Department of Maternal and Child Health, School of Public Health, Tongji Medical College, Huazhong University of Science and Technology, Wuhan, China; 2 Department of Social Medicine and Health Management, School of Public Health, Wuhan University, Wuhan, China; 3 Center for Injury Research and Policy, The Research Institute at Nationwide Children's Hospital, The Ohio State University, Columbus, Ohio, United States of America; 4 Department of Epidemiology and Statistics, School of Public Health, Wuhan University, Wuhan, China; Nottingham University, United Kingdom

## Abstract

**Objectives:**

We compared the patterns of medically attended injuries between children with and without disabilities and explored the residential environment risks in five counties of Hubei Province in the People's Republic of China by a 1∶1 matched case-control study based on the biopsychosocial model of the International Classification of Functioning, Disability and Health – ICF.

**Methods:**

1201 children aged 1–14 with disabilities and 1201 their healthy counterparts matched as having the same gender, same age, and lived in the same neighborhood were recruited in our study. Characteristics of injuries in the past 12 months were compared between children with and without disabilities. The associations among disability status, home environment factors and injuries were examined in logistic regression analysis taking into account sociodemographic factors.

**Results:**

Children with disabilities had a significantly higher prevalence of injury than children without disabilities (10.2% vs. 4.4%; *P*<.001). The two groups differed significantly in terms of number of injury episodes, injury place and activity at time of injury. Falls were the leading mechanism of injury regardless of disability status. Most of the injury events happened inside the home and leisure activities were the most reported activity when injured for both groups. The univariate OR for injury was 4.46 (2.57–7.74) for the disabled children compared with the non-disabled children. Disabled children whose family raised cat/dog(s) were 76% more likely to be injured during the last 12 months (OR = 1.76; 95% CI = 1.02, 3.02),comparing with those whose family did not have any cat/dog. And for children without disabilities, those whose family had cat/dog(s) were over 3 times more likely to having injuries comparing with those whose family did not have any cat/dog.

**Conclusions:**

Children with disabilities had a significantly increased risk for injury. Interventions to prevent residential injury are an important public health priority in children with disabilities.

## Introduction

It is estimated that 10% of children globally (approximately 200 million) suffer from some kind of disability, and most of whom live in low- and middle-income countries [1], [2]. The burden of childhood disability in these areas remains relatively unrecognized. Despite rising interest in child disability, little is known about the frequency and situation of children with disabilities in the developing world [3]. In China, with the improvements in health care services and a decrease in child mortality, disabled children have been more likely to survive in greater numbers and come much more than before. According to a national survey, approximately 3.87 million children younger than 14 years of age in China had a disability [4]. Children with disabilities have long-term physical, mental, intellectual or sensory impairments, which interacting with attitudinal and environmental barriers may hinder a child's full and effective participation in society on an equal basis with other children [3], [5]. Children who have disabilities have much higher costs resulting from disability–such as costs associated with medical care or assistive devices, or the need for personal support and assistance–and thus often require more resources than do their healthy peers. However, there is a paucity of research about the child disability, despite the fact that a large burden of disability on child development, economics, and family life in China.

Prevention of secondary conditions among children with disabilities is an emerging global health priority. Children with disabilities are at higher risk for “secondary conditions” including injuries [6]–[8]. Many studies investigated injuries among children with disabilities and reported that children with disabilities have an increased risk for injuries compared with children without disabilities [9]–[12]. Evidences indicated that this risk might be due to characteristics in children that result from their disabilities, such as physical limitations, cognitive impairments, anti-social behavior, or inability to adapt to the environment [13]–[17]. Family and environmental factors is another proposed explanations for childhood injuries [18]–[20]. The home environment is considered as one of the most essential contextual factor that contributes to young children's unintentional injury [21]–[23]. Although numerous studies have reported rates of injury in children with disabilities, there is a major information gap in China on the magnitude of the problem and possible implicated risk factors. Therefore, we conducted a 1∶1 matched case-control study in order to identify the potential risk factors of medically-attended injuries within this vulnerable Chinese pediatric population.

## Methods

### Ethics statement

Informed written consent was obtained from a legal guardian of each child involved in our study. This study was approved by the Institutional Review Board of Tongji Medical College, School of Public Health, Huazhong University of Science & Technology.

### Data Source

This study is based on the registry database of China Disabled Persons' Federation (CDPF). CDPF is the official agency for individuals with disabilities. The registry database is set up to monitor the total number of persons with disabilities and is used for providing services. Individuals with disability may qualify for certain government funded services. A person needs to be evaluated by a certified physician using the official disability assessment criteria in order to receive such benefits. Individuals who meet criteria will be issued an official certificate that lists the type and the severity of the disability he/she has. Their information is then entered into the registry database by the CDPF's county level office for administrative management. We reviewed the registry database of persons with disabilities in five counties that were randomly selected in Hubei Province in central China.

Disabled children aged 1 to 14 years were selected in our study, and for those who agreed to participate in the study, a 25–35 minutes face-to-face interview was conducted with a legal guardian of the child. For every disabled child, we matched a normal nondisabled child (same gender and age, and lived in the same neighborhood).

Both disabled children and their healthy peers were interviewed using the same questionnaire which was developed together by researchers from Tongji Medical College, School of Public Health of Wuhan University and the Center for Injury Research and Policy, the Research Institute at Nationwide Children's Hospital, the Ohio State University College of Medicine. The questionnaire included sociodemographic information and health-related questions, and besides, there are questions about limitations in daily activities. Factors about the home and neighborhood physical environment that may contribute to injury risk and details about medically-attended injuries occurred in the previous 12 months were also collected. The questionnaire was first piloted in a small group of children in one of the selected five counties. Questionnaire was finalized using feedback from the pilot study.

### Disability Classification

The study adopted the language and concepts of the International Classification of Functioning, Disability and Health (ICF) – a conceptual framework formulated by the World Health Organization (WHO) to define disability. The ICF aims to provide standardized terminology for epidemiological studies to achieve comparability of data at national and international level. This framework describes the etiology of decrements in functioning and disability not only in association with individual health conditions or abilities but also in association with two contextual factors, environment and personal factors, such as social support, culturally influenced perceptions of disability, social background and access to education [24], [25]. Respondents were asked several questions to determine whether their child was limited in any way when engaging in some age-appropriate activities. Responding “yes” to at least one of the following four questions indicated the child was limited in any way: (1) whether the child was limited in any way in any activities because of any impairment or health problem; (2)whether the child received special education or early intervention services, (3)whether the child needed the help of adults with personal care needs such as feeding and bathing because of a physical, mental, or emotional problem; (4)whether the child needed some special equipments or assistive devices because of a health problem. If the child was limited, respondents were given 17 fixed condition categories to report what impairment or health problem caused the child's limitation.

### Injury Definition

An injury case was defined as an event happened to a child in the previous 12 months that resulted in medical attention. Respondents were asked to report any medically–attended injury episode that occurred during the 12 months prior to the interview. We analyzed both the number of injured children and the number of injury episodes for each child. Detailed information were collected about the most recent injury episode, including the cause of injury, type of injury, body part injured, injury place, and activity at time of injury. We used the primary cause in the process of injury event to describe the external cause. For instance, if a child collided with an object first and then fell, the primary cause of injury was a collision. In our design, the disabling condition was viewed as a risk factor for injury, therefore, a child must have the disabling condition(s) for at least 12 months prior to the interview to ensure that the child's disability presented before the injury of interest occurred.

### Home Environment

Hazards such as unlocked storage of fireworks, cats/dogs raising, slippery floor, opening burner or brazier are common and might elevate the risk of childhood injury in China. In this study, we collected 8 physical risks in the home that probably play a role in injury risk for children. Questions about safety hazards of the children' homes included: (Q1) “Whether the home has any cat/dog?” (Q2) “Whether the home has fireworks stored unlocked?” (Q3) “Whether the family uses open burner or brazier in winter to keep warm?” (Q4) “Whether the floor is anti-slippery?” (Q5) “Whether the indoor light is bright?” (Q6) “Whether the heat source is easy for child to get?” (Q7) “Whether the child has any BB gun?” (Q8) “Whether the home has safety window grille?”

### Sociodemographic Variables

We included gender, age, parent's education, number of family members, family income and time of being cared per-day that might affect the association between disability status and injury risk. The family members include the child, the child's parents/siblings/grand-parents/father's sister or brother who is not married. The time of being cared per day refers to the average time that child who is within the range of his/her primary caregiver's vision per day. RMB is the official currency of the People's Republic of China. One RMB is approximately equal to U.S.$ 0.157.

### Statistical Analysis

Data analyses were conducted using SAS statistical software. We first compared the prevalence of injuries between the disabled and nondisabled children by gender, age, parent's education, number of family members, average time of being cared per-day, and family income per month. We used the Chi-square test to determine the association between injury status and disability status by the sociodemographic variables mentioned above. By calculating distribution percentage (%) and 95% confidence intervals of injuries, we also made a comparison about the characteristics of injury episodes between children with and without disabilities including injury times, cause, type, body part injured, injury place and activity at time of injury. The distributions of injuries status in the sampled children were also calculated by the above sociodemographic variables. Injury Rate among children with and without disabilities by home environment factors was calculated. Finally, logistic regression model was used to assess the injury risk for sampled children by disability status, sociodemographic characteristics and home environment factors in univariate model. Children with and without disabilities were analyzed separately in multivariate model. We tested whether basic sociodemographic factors might influence the presence or absence injury risks at the child's home. In all analyses, differences at *P*<0.05 from the significant test were considered statistically significant.

## Results

1379 children with disabilities were recorded in registry database in the selected five counties and the overall final response rate was 87.1% in this study. A total of 1201 disabled children aged 1 to 14 years and 1201 their healthy controls were included in our study. The sample included 807 boys and 394 girls in both groups of children with and without disabilities. The mean age of the study sample was 6.9±3.5 years. Table 1 shows the prevalence of medically-attended injuries occurred during the 12 months prior to interview for children with and without disabilities, stratified by sociodemographic characteristics. Children with disabilities had substantially higher prevalence of injury than children without a disability (10.2% vs. 4.4%, *P*<.001). This trend was consistent throughout the most sociodemographic categories. However, for children aged 1- to 4-year-olds and 11- to 14-year-olds; for children whose parents' highest education was middle school or less; and for children whose family income per month was less than 1000 RMB and 5000 RMB or higher, the injury prevalence did not differ significantly between children with and without disabilities (*P*>.05). Nevertheless, the non-significant differences for variables age, education, and income all showed a trend toward becoming significant.

**Table 1:** Prevalence of a Medically Attended Injury among Children With and Without Disabilities, by Sociodemographic Characteristics in Hubei, China.

Characteristics	Children with disabilities		Children without disabilities		*P-value*
	N	Injured N (%)	N	Injured N (%)	
**Total**	1201	123(10.2)	1201	53(4.4)	0.000
**Gender**					
Boy	807	87(10.8)	807	36(4.5)	0.000
Girl	394	36(9.1)	394	17(4.3)	0.007
**Age (years)**					
1–4	359	29(8.1)	359	19(5.3)	0.135
5–10	598	70(11.7)	598	21(3.5)	0.000
11–14	244	24(9.8)	244	13(5.3)	0.060
**Parent's education** a					
Middle school or less	299	24(8.0)	148	6(4.1)	0.114
High school	778	83(10.7)	748	30(4.0)	0.000
Undergraduate degree or higher	124	16(12.9)	315	17(5.4)	0.010
**Number of family members**					
1–3	233	28(12.0)	353	22(6.2)	0.014
4–5	661	79(12.0)	620	28(4.5)	0.000
6 or more	307	16(5.2)	228	3(1.3)	0.016
**Average time of being cared per day**					
<30%	81	10(12.3)	256	15(5.9)	0.052
30–59%	163	24(14.7)	332	15(4.5)	0.000
60–89%	418	42(10.0)	444	19(4.3)	0.001
>90%	539	47(8.7)	169	4(2.4)	0.005
**Family income per month**					
Less than 1000 RMB	186	16(8.6)	65	5(7.7)	0.820
1000–3000 RMB	563	65(11.5)	413	14(3.4)	0.000
3000–5000 RMB	389	35(9.0)	513	22(4.3)	0.004
5000 or higher	63	7(11.1)	210	12(5.7)	0.140

a Parent's education was defined as the highest level of education achieved by either the child's mother or father.

Table 2 presents a comparison of the characteristics of injury episodes among the sampled children with and without disabilities. The two groups differed significantly with respect to injury times, injury place, and activity at time of injury. The disabled children were more likely to experience 3 or more times of injury than the normal healthy children (19.5% vs. 3.8%). The most frequent cause of injury for both groups was falls (44.7% vs. 34.0%). Children with disabilities were more frequently hurt by fire/flames/heat than children without disabilities (15.4% vs. 7.5%), however, traffic-related injuries occurred more often among the nondisabled children (17.0% vs. 6.5%). For type of injury, both groups reported open wounds and superficial injury most frequently, but burns were more common for children with disabilities. Most of the injury events happened inside the home and leisure activities were the most reported activity when injured for both groups. Different things were that nondisabled children were more commonly injured while attending school (18.9%), whereas those children with disabilities were more commonly sleeping or resting (11.4%). Both groups reported head/neck regions and lower extremities (leg/knee) were the most common body parts injured.

**Table 2:** Characteristics of Injuries in Sampled Chinese Children With and Without Disabilities, Hubei, China.

Characteristic	Children with disabilities		Children without disabilities	
	Injured No.	% (95% CI)	Injured No.	% (95% CI)
Number of injury episodes*				
1	89	72.4(64.1, 79.9)	48	90.6(81.3, 96.9)
2	10	8.1(4.0, 13.6)	3	5.7(1.1, 13.4)
3 or more	24	19.5(13.0, 27)	2	3.8(0.4, 10.5)
**Cause of injury**				
Fall	55	44.7(36.1, 53.5)	18	34.0(21.9, 47.1)
Fire/flames/heat	19	15.4(9.6, 22.3)	4	7.5(2.1, 16.1)
Animal/insect bite	17	13.8(8.3, 20.5)	6	11.3(4.3, 21.1)
Struck by a person or object	13	10.6(5.8, 16.6)	10	18.9(9.6, 30.4)
Other cause	11	8.9(4.6, 14.6)	6	11.3(4.3, 21.1)
Traffic injury	8	6.5(2.8, 11.5)	9	17.0(8.2, 28.2)
**Type of injury**				
Open wound	39	31.7(23.8, 40.2)	9	17.0(8.2, 28.2)
Superficial injury	35	28.5(20.9, 36.7)	20	37.7(25.3, 51.1)
Burn	19	15.4(9.6, 22.3)	3	5.7(1.1, 13.4)
Others^a^	15	12.2(7.0, 18.5)	9	17.0(8.2, 28.2)
Fracture	10	8.1(4.0, 13.6)	7	13.2(5.5, 23.5)
Sprain/strain	5	4.1(1.3, 8.3)	5	9.4(3.1, 18.7)
Injury place*				
Inside home	67	54.5(45.6, 63.2)	16	30.2(18.7, 43.1)
Outside home	22	17.9(11.6, 25.1)	16	30.2(18.7, 43.1)
Other location^b^	21	17.1(11.0, 24.2)	9	17.0(8.2, 28.2)
Kindergarten/school	13	10.6(5.8, 16.6)	12	22.6(12.5, 34.8)
Activity at time of injury*				
Leisure activity	79	64.2(55.6, 72.4)	29	54.7(41.3, 67.8)
Other activity^c^	18	14.6(9.0, 21.4)	10	18.9(9.6, 30.4)
Sleeping/resting	14	11.4(6.4, 17.6)	2	3.8(0.4, 10.5)
Eating/drinking	8	6.5(2.8, 11.5)	2	3.8(0.4, 10.5)
Attending school	4	3.3(0.9, 7.1)	10	18.9(9.6, 30.4)
**Body part injured**				
Head/neck	48	39.0(30.6, 47.8)	20	37.7(25.3, 51.1)
Lower extremities	36	29.3(21.6, 37.6)	16	30.2(18.7, 43.1)
Upper extremities	25	20.3(13.7, 27.9)	15	28.3(17.1, 41.1)
Other part^d^	14	11.4(6.4, 17.6)	2	3.8(0.4, 10.5)

a Other injury includes internal injury of abdomen or pelvis; complicated and unspecified; injury to blood vessels, coma, cold injury, and other.

b Other location includes health care center, trade or service area, street or highway, neighbor's house, river/pond, sidewalk, farm, orchard, and other.

c Other activity includes doing exercise, care from another person, bathing, beating dog/cat, setting off fireworks, stirring up hornet's nests, walking, cooking, having a warm, and other.

d Other part includes chest, abdomen, pelvis, spine, buttock, low back, unspecified, Multi-body parts and other.

In table 3, we stratified the sample children into three groups based on the location of injury occurrence: residential injury, non-residential injury and no injury. Residential and non-residential injuries refer to those who experienced an injury inside the home and in all other locations, respectively. Table 3 presents the proportion (%) and 95% CI of medically-attended injury by sociodemographic characteristics. The rates of non-fatal residential and non-residential injury were 3.5% and 3.9%, respectively. Disability status, parent's education and number of family members were significantly associated with residential injury. However, disability status and parent's education both were not associated with non-residential injury. Gender, age, family income and average time of being cared per day were not associated with injury in this analysis.

**Table 3:** Distribution of Medically Treated Injuries in the Sampled Chinese Children by Sociodemographic Characteristics.

Characteristic	Injury at home		Other Injury		No Injury	
	N	% (95% CI)	N	% (95% CI)	N	% (95% CI)
**Total**	83	3.5	93	3.9	2226	92.6
**Disability status** *						
No Disability	16	1.3(0.8, 2.1)	37	3.1(2.2, 4.1)	1148	95.6(94.4, 96.7)
Disability	67	5.6(4.4, 6.9)	56	4.7(3.5, 5.9)	1078	89.8(88.0, 91.4)
**Parent's education** *						
Middle school or less	14	3.1(1.7, 4.9)	16	3.6(2.1, 5.5)	417	93.3(90.8, 95.4)
High school	62	4.1(3.1, 5.1)	51	3.3(2.5, 4.3)	1413	92.6(91.2, 93.9)
Undergraduate degree or higher	7	1.6(0.7, 3.0)	26	6.1(4.0, 8.5)	396	92.3(89.6, 94.6)
**Number of family members** *						
1–3	18	3.1(1.8, 4.6)	32	5.5(3.8, 7.4)	536	91.5(89.1, 93.6)
4–5	57	4.4(3.4, 5.6)	50	3.9(2.9, 5.0)	1174	91.6(90.1, 93.1)
6 or more	8	1.5(0.6, 2.7)	11	2.1(1.0, 3.4)	516	96.4(94.7, 97.8)
**Average time of being cared per day**						
<30%	8	2.4(1.0, 4.3)	17	5.0(3.0, 7.6)	312	92.6(89.5, 95.1)
30–59%	14	2.8(1.6, 4.5)	25	5.1(3.3, 7.2)	456	92.1(89.6, 94.3)
60–89%	32	3.7(2.6, 5.1)	29	3.4(2.3, 4.7)	801	92.9(91.1, 94.5)
>90%	29	4.1(2.8, 5.7)	22	3.1(2.0, 4.5)	657	92.8(90.8, 94.6)
**Family income per month**						
Less than 1000 RMB	10	4.0(1.9, 6.7)	11	4.4(2.2, 7.3)	230	91.6(87.9, 94.7)
1000–3000 RMB	38	3.9(2.8, 5.2)	41	4.2(3.0, 5.5)	897	91.9(90.1, 93.5)
3000–5000 RMB	30	3.3(2.3, 4.6)	27	3.0(2.0, 4.2)	845	93.7(92.0, 95.2)
5000 or higher	5	1.8(0.6, 3.8)	14	5.1(2.8, 8.1)	254	93.0(89.7, 95.7)
**Age**						
1–4	31	4.3(3.0, 5.9)	17	2.4(1.4, 3.6)	670	93.3(91.4, 95.0)
5–10	36	3.0(2.1, 4.1)	55	4.6(3.5, 5.9)	1105	92.4(90.8, 93.8)
11–14	16	3.3(1.9, 5.0)	21	4.3(2.7, 6.3)	451	92.4(89.9, 94.6)
**Gender**						
Boy	52	3.2(2.4, 4.1)	71	4.4(3.5, 5.5)	1491	92.4(91.0, 93.6)
Girl	31	3.9(2.7, 5.4)	22	2.8(1.8, 4.1)	735	93.3(91.4, 94.9)

* *P*<.05.

Table 4 presents the home injury rate among children with and without disabilities by home environment factors. Eight risks that might be present in the physical environment of child's home were collected. As shown in table 4, there was significant residential injury difference for variable about whether having cat/dog in children with disabilities. The rest of safety measures of homes were not associated with injuries occurred at the home for both groups.

**Table 4:** Injury Rate Among Children With and Without Disabilities by Home Environment Factors.

Characteristics	Children with disabilities		Children without disabilities	
	N	Inj % (95% CI)	N	Inj % (95% CI)
**Having cat/dog**				
No	886	4.7(3.4, 6.2)	923	1.0(0.4, 1.7)
Yes	315	7.9(5.2, 11.2)*	278	2.5(1.0, 4.7)
**Fireworks stored unlocked**				
No	1139	5.5(4.3, 6.9)	1131	1.4(0.8, 2.2)
Yes	62	6.5(1.7, 13.9)	70	0.0
**Open burner or brazier**				
No	656	4.6(3.1, 6.3)	802	1.0(0.4, 1.8)
Yes	545	6.8(4.8, 9.1)	399	2.0(0.9, 3.6)
**Anti-slippery floor**				
No	638	5.5(3.9, 7.4)	474	1.5(0.6, 2.8)
Yes	563	5.7(3.9, 7.8)	727	1.2(0.6, 2.2)
**Bright indoor light**				
No	179	3.9(1.6, 7.3)	79	1.3(0.1, 4.9)
Yes	1022	5.9(4.5, 7.4)	1122	1.3(0.8, 2.1)
**Easy to get heat source**				
No	794	6.2(4.6, 8.0)	759	1.3(0.6, 2.3)
Yes	407	4.4(2.6, 6.6)	442	1.4(0.5, 2.8)
**Having any BB gun**				
No	930	6.0(4.6, 7.6)	926	1.3(0.7, 2.1)
Yes	271	4.1(2.0, 6.7)	275	1.5(0.4, 3.2)
**Having safety window grille**				
No	618	5.2(3.6, 7.1)	432	1.9(0.8, 3.3)
Yes	583	6.0(4.2, 8.1)	769	1.0(0.5, 1.9)

* *P*<.05.

Table 5 shows the ORs for medically-attended injuries happened at home by disability status, sociodemographic characteristics and home environment factors. In univariate analysis, the OR for injury was 4.46 (2.57–7.74) for children with disabilities compared with children without disabilities. The child whose parent's highest education was high school, whose family had 4 to 5 members, cat/dog(s) or opening burner/brazier was at the highest risk for injury. The multivariate OR for injury was 3.36 (1.48–7.61) for the disabled children who had 4 to 5 family members compared with those who had 6 or more family members after controlling for sociodemographic and home environmental factors. And for the disabled children whose family raised cat/dog(s) were over 75% more likely to be injured during the last 12 months (OR = 1.76; 95% CI = 1.02, 3.02), comparing with those whose family did not have any cat/dog. For children without disabilities, those whose family had cat/dog(s) were over 3 times more likely to having injuries comparing with those whose family did not have any cat/dog.

**Table 5:** Logistic Regression Analysis of Medically Treated Home Injuries by Disability Status, Sociodemographic Characteristics and Home Environment Factors.

Variables	Univariate Model	Multivariate Full Model	
		Children with disabilities	Children without disabilities
	OR 95% CI	OR 95% CI	OR 95% CI
**Disability status**			
No Disability	1		
Disability	4.46(2.57, 7.74)*		
**Average time of being cared per day**			
<30%	1.72(0.78, 3.81)	0.67(0.23, 1.97)	0.24(0.02, 3.25)
30–59%	1.56(0.71, 3.42)	0.82(0.29, 2.34)	0.60(0.10, 3.76)
60–89%	1.20(0.50, 2.89)	0.75(0.23, 2.46)	1.66(0.36, 7.63)
>90%	1	1	1
**Gender**			
Boy	1.21(0.77, 1.90)	0.91(0.53, 1.55)	0.98(0.31, 3.09)
Girl	1	1	1
**Age**			
1–4	1.31(0.71, 2.40)	1.27(0.56, 2.88)	4.36(0.75, 25.38)
5–10	0.91(0.51, 1.67)	1.19(0.57, 2.48)	0.54(0.11, 2.58)
11–14	1	1	1
**Number of family members**			
1–3	2.16(0.93, 5.03)*	2.97(1.13, 7.76)*	4.44(0.45, 44.06)
4–5	3.13(1.48, 6.61)*	3.36(1.48, 7.61)*	5.07(0.62, 41.35)
6 or more	1	1	1
**Parent's education^a^**			
Middle school or less	1.90(0.76, 4.76)	0.90(0.27, 3.05)	1.68(0.11, 26.56)
High school	2.48(1.13, 5.47)*	1.33(0.45, 3.90)	4.95(0.82, 29.92)
Undergraduate degree or higher	1	1	1
**Family income per month**			
Less than 1000 RMB	2.21(0.74, 6.56)	1.52(0.28, 8.17)	0.48(0.04, 6.36)
1000–3000 RMB	2.15(0.84, 5.52)	2.03(0.43, 9.58)	0.32(0.06, 1.85)
3000–5000 RMB	1.81(0.69, 4.70)	1.93(0.41, 9.05)	0.49(0.10, 2.34)
5000 or higher	1	1	1
**Having cat/dog**			
No	1	1	1
Yes	2.01(1.30, 3.21)*	1.76(1.02, 3.02)*	3.21(1.12, 9.23)*
**Fireworks stored unlocked**			
No	1	1	
Yes	0.85(0.31, 2.36)	1.01(0.34, 2.98)	
**Open burner or brazier**			
No	1	1	1
Yes	1.86(1.20, 2.89)*	1.29(0.76, 2.22)	1.81(0.60, 5.42)
**Anti-slippery floor**			
No	1.21(0.77, 1.87)	1.03(0.60, 1.76)	1.20(0.40, 3.60)
Yes	1	1	1
**Bright indoor light**			
No	0.89(0.43, 1.87)	0.72(0.30, 1.69)	1.15(0.13, 10.21)
Yes	1	1	1
**Easy to get heat source**			
No	0.74(0.46, 1.20)	1.35(0.75, 2.42)	0.85(0.26, 2.79)
Yes	1	1	1
**Having any BB gun**			
No	0.76(0.43, 1.33)	1.70(0.84, 3.46)	0.94(0.27, 3.30)
Yes	1	1	1
**Having safety window grille**			
No	0.83(0.54, 1.30)	0.87(0.52, 1.46)	1.90(0.64, 5.61)
Yes	1	1	1

* *P*<.05.

## Discussion

Our study is possibly the first study that investigated non-fatal unintentional injury risk among children with disabilities in China based on the biopsychosocial model of the International Classification of Functioning, Disability and Health – ICF. In the present study, we found a strong association between disability status and injury in this sample of Chinese pediatric population. Compared with children without disabilities, children with disabilities were over 4 times more likely to be injured in the previous 12 months. We also found that above half of injuries among children with disabilities occurred inside the home, and nearly 50% injuries were caused by falls.

Xiang and colleagues found that children with developmental disabilities or chronic medical conditions were at a higher risk for injury than were children without those conditions [11]. Sherrard et al. conducted a longitudinal study in a group of intellectually challenged individuals in Australia and found the rate of injury hospitalizations in these individuals to be twice that of the general population.^14^Other studies also indicated that children with disabilities were over two times more likely to experience an injury that needs medical attention compared with their healthy controls [26], [27]. Findings from this study are consistent with these studies using parent-reported or caregiver-reported data on the association between injury and disability.

Consistent with results from previous studies, we found that a higher percentage of injuries among children with disabilities was caused by falls and occurred inside the home. In addition, a higher proportion of children with disabilities were injured when they are sleeping or resting [10]–[12], [17], [28]–[30]. These findings suggested that the strategies to minimize the risk of falls and to prevent injuries occurred in residential areas among children with disabilities are of great importance. Children with disabilities suffered residential injuries more often than children without a disability probably because they are less likely to participate in activities outside the home compared with their healthy peers due to their activity limitation and participation restrictions caused by their disability.

Extraordinary numbers of children worldwide suffer from preventable injuries that occur within their home [31]. It is estimated that approximately 12 million non-fatal, medically treated injuries occur in and around the home annually [32], [33]. In this study, we found that above 50% pediatric injuries happened inside the home in this sample of Chinese children. There are evidence that falls, burns, unintentional cuts, poisonings and animal bites are especially common for young children in low- and middle income countries, and that these injuries often happen at home [31], [34], [35]. Home environment is one of the most significant contextual factors of injury risk for young children [22]. Hazards such as open fires, owning cats/dogs, fireworks stored unlocked, and slippery floor are very common in children's homes in China, and all these contribute to young children's high levels of unintentional injury risk. However, in the present study, we only found that child whose family had cat/dog(s) was over 3 times more likely to be injured, compared with child whose family did not have any cat/dog during the last 12 months after adjustment for sociodemographic factors and disability status. The rest of 7 physical home environmental risks did not have significant association with pediatric injuries. Future work should explore these risks in larger samples, and identify whether and how they might translate into actual injury incidence and examine which disabilities are associated with the greater risk of injury. Although some studies suggest that children from larger families are at higher risk of injury [34], [36], our findings suggested a reversed association which was consistent with a previous research that children with less family members are at higher risk of injury [37]. This is probably because there are strong family ties and a phenomenon that older children and other family members would watch over the younger child in Chinese family.

Like all research, our study has some limitations. First, the definition of disability in our study was based on the new ICF which is quite broad, and includes a very broad range of children with a very broad range of types of disabilities. Future study might consider specific types of disabilities individually (e.g., mental retardation, wheelchair-users, etc). Second, our analysis results are limited by a small sample size because of the comparatively low prevalence of injury among children with and without disabilities (10.2% and 4.4%, respectively). The injury definition in our study is different from the previous studies in China. Injuries defined in the previous studies included: (1) medically attended injury; or (2) injury leading children off school more than half a day; or (3) first-aid by family members or teachers or friends [38]. Previous studies reported that the prevalence of injury among healthy children aged 1 to 14 was 5.3∼11.3% [39], [40]. In our study, the prevalence of injury (4.4%) among nondisabled children is lower than those reported in previous studies. However, if we counted injuries treated by family members or teachers or friends, the prevalence of injury among children with and without disabilities is 17.5% and 8.6%, respectively. In order to compare our findings with previous studies conducted around the world, we choose to report only medically treated injuries in our study. Another reason of low prevalence of injury both among children with and without disabilities is that caregivers' medical care-seeking behavior when their children injured is extremely low in China, especially in rural areas where medical service is not well developed. Unfortunately, a larger percent of children with disabilities are in rural counties in China so that the majority of respondents in our study are from those underdeveloped areas. Third, there was no information about validity of self-report of time per day that children were supervised, and some of the other measures used in the study. This limitation is inherent in the self-reported surveys. And also, there were no data on self-inflicted injuries. Injury were reported from primary caregiver and were not validated by medical records is another limitation of our study. Previous studies indicated that underestimation of injury episodes were likely to happen when using a 12-month recall period [41], [42]. Information bias is also possible if parents of children with disabilities systematically reported injuries differently than parents of normal children. Parents of children with disabilities may tend to request medical attention more often than other parents when an injury happens to their child. Further research is needed to identify how disability status influences recall bias in injury reporting by caregivers. Finally, one would expect to see an association between age and injury risk because children have different level of injury risk at different developmental stages. Our study did not find an association between developmental age and injury rate. This could be due to small sample size in age subgroups. In addition, our results indicated non-significant differences with respect to certain subgroups of age, education, and income between children with and without disability, which may due to the small numbers for each of these. A larger study would probably find statistical differences.

Despite these limitations, our findings have implications for injury prevention targeting children with disabilities. Parents and other caretakers of children with disabilities should receive additional education on home safety and injury prevention. Injury prevention needs to be emphasized for the sake of reducing the excess incidence of injury among children with disabilities. In addition, nurses, pediatricians and other healthcare professionals should be aware of the significantly high risk for injury in children with disabilities. They could plan an important role in educating parents and caregivers about safety and injury prevention when they provide medical services to children with disabilities. Furthermore, in view of a higher proportion of residential injuries, intervention strategies need to be initiated to improve the safety of children's home environments. Although every child is susceptible to unintentional injury, children with disabilities not only have a specific vulnerability, but they are more likely to stay at home and therefore are at significant higher risk of residential injuries than children without disabilities. Interventions to prevent residential injuries are an important public health priority in children with disabilities in the People's Republic of China.
